# Transmission ratio distortion in families from the Framingham Heart Study

**DOI:** 10.1186/1471-2156-4-S1-S48

**Published:** 2003-12-31

**Authors:** Andrew D Paterson, Lei Sun, Xiao-Qing Liu

**Affiliations:** 1 Program in Genetics and Genomic Biology, The Hospital for Sick Children, Toronto, Ontario, Canada; 2 Department of Public Health Sciences, University of Toronto, Toronto, Ontario, Canada

## Abstract

**Background:**

One implicit assumption in most linkage analysis is that live-born siblings unselected for a phenotype do not share alleles greater than the Mendelian expectation at any particular locus. However, since most families are recruited for genetic studies because of the presence of disease, there is little data available to confirm that this is the case. We hypothesized that loci that behave in a non-Mendelian fashion could be identified using genotype data from the Framingham Heart Study families. We tested the hypothesis that live-born sibs, either stratified by or irrespective of gender, demonstrate excess sharing of alleles on the autosomes, i.e., transmission ratio distortion. Multipoint linkage analysis of siblings either according to gender or not was performed using an allele-sharing method. Such observations may have implications for the mapping of loci for complex disease and quantitative traits in human pedigrees.

**Results:**

No results that reached genome-wide significance were observed. However, four regions demonstrated excess sharing of alleles at *p *< 0.002 when sibships were stratified by gender-three of which were present in males. Of note, a female-specific locus co-localized with region that is linked to mean systolic blood pressure in the same families. In addition, three other regions demonstrated excess sharing of alleles in sibships irrespective of gender, including a region on chromosome 10p14-p15 (*p *= 7.5 × 10^-4^).

**Conclusion:**

Although no loci meeting genome-wide significance were detected to demonstrate transmission ratio distortion, loci with suggestive evidence for linkage were detected. These may have implications for the mapping of susceptibility loci for complex disease in human pedigrees.

## Background

"No substantial study of normal sib-pairs has been undertaken, making this family of surveys one of the largest undertaken in the absence of controls" [1]. The two main motivations for this study are reports of transmission ratio distortion at a number of loci in humans and the often nonsignificant results of genetic linkage studies of complex diseases.

Transmission ratio distortion (TRD) has been defined as "a statistically significant departure from the Mendelian inheritance ratio expected regardless of the cause" [2]. There are a number of reports indicating that TRD may be present at specific regions in the human genome (reviewed in [2]), but many are based on families with multiple individuals affected with a particular disease, making it difficult to determine whether such phenomena are somehow related to the disease, or are a general occurrence. In some cases, families not recruited for disease have been used, but some of these, such as the Centre d'Etude du Polymorphisme Humain (CEPH) reference families, were specifically ascertained for their large sibship sizes and the availability of grandparents. Use of such pedigrees may reduce the proportion of families demonstrating TRD compared with the general population (see Discussion). Furthermore, attempts to identify TRD loci have not been performed in a systematic genome-wide fashion: regions have been selected either for the presence of putative disease susceptibility loci (e.g., [3]); or loci that contain genes that have been documented to demonstrate genomic imprinting [4,5].

At certain TRD loci, the effect may be sex-specific [3-5], and this may have important implications for the mapping of loci for common complex disease. Sex differences in the age-specific incidence are a general feature of common complex diseases. Obviously, certain diseases either solely (e.g., ovarian cancer, prostate cancer), or predominantly (e.g., breast cancer, systemic lupus erythematosus, autism) affect individuals of one gender. Due to theoretical considerations, most genetic mapping studies of common complex diseases have employed affected sib pairs only [6]. In fact, unaffected siblings are rarely recruited or genotyped since it has been argued that unless the prevalence of the trait is high, they do not provide sufficient linkage information to justify study [6]. Thus any locus that produces TRD in a sex-specific fashion would be expected to also demonstrate evidence for linkage to a disease in which the majority of affected individuals were of that gender-and this would likely be spurious linkage [7]. There have been reports of sex-specific autosomal loci for some complex diseases, but it has generally not been possible to determine whether these are spurious linkages with disease resulting from sex-specific TRD because of the paucity of genotyped unaffected sibs. Finally, genetic linkage studies of complex diseases have generally produced nonsignificant results using genome-wide criteria. This makes it difficult to distinguish true linkage signals from noise. Noise from biological phenomenon such as TRD could therefore potentially mislead disease gene mapping efforts.

The Framingham families represent a valuable resource for the study of TRD because they are relatively ethnically homogeneous (predominantly White), and were not recruited for the presence of disease or trait value. The only reasons that families were included in the genetic studies was based on their age being 28-62 years for the original cohort and 12-58 years for the offspring cohort at recruitment, being alive when DNA was obtained (in the late 1980s and early 1990s), and their relatively large size (although the exact criteria used to determine size were not specified). Results from these families will help to determine whether concerns about TRD are of general concern for the mapping of susceptibility loci for complex diseases.

## Methods

Our primary interest is to identify autosomal TRD loci in the Framingham Heart Study families. We hypothesized that such loci could act either in a sex-specific or nonspecific fashion. Ethics approval was obtained from the Hospital for Sick Children Research Ethics Board (#2002/116). Genotype data from 398 autosomal microsatellite markers in 1702 individuals from 330 pedigrees were used. To test for sex-specific TRD, families were divided into nuclear families containing two or more genotyped same-sex siblings. There were 222 sibships with ≥2 genotyped female sibs and 201 such male sibships (called female and male families, respectively). In the female families, female siblings were coded as affected. Similarly, in the male families, male siblings were coded as affected. Siblings of the opposite sex, when present, were kept in the family to improve estimates of allele sharing between siblings, but were coded as unaffected. Marker allele frequencies were counted from the founders in the male and female families separately.

To investigate TRD that is not sex-specific, nuclear families with two or more genotyped siblings were generated irrespective of gender. There were 459 such families. Any genotyped sib was coded as affected. For both the sex-specific and nonspecific data, multipoint nonparametric linkage analysis was performed using ALLEGRO v. 1.1b [8] using the exponential model, S_all _scoring function, and the recommended family weighting scheme of power: 0.5. *p*-Values were obtained using normal approximation.

## Results

In Table 1 the number of siblings per family with genotype data for each of the three groups of families are indicated. The majority of families have two siblings in all three of the groups. Table 2 summarizes the linkage analysis results for any region with *p *< 0.002 for the sex-specific analysis. The results for the male and female families are provided for comparison. No results reached the criteria for suggestive linkage [9]. Of note, three of the four loci were identified in male sibships, although the number of sibships was smaller than the female families (Table 1). Also, information content was lower in male sibships than female sibships at the regions showing linkage to males.

When we tested for the hypothesis that TRD would act in non-sex-specific fashion we identified three regions with *p *< 0.002 (Table 3). None of these regions overlapped with the sex-specific regions identified in Table 2. The most significant result observed was at 14 cM from pter on chromosome 10 in the sex-averaged genetic map, peaking between markers GATA88F09 and AFM063xf4 (also known as D10S1435 and D10S189, respectively). The result at this region did not formally reach the criteria for suggestive linkage (*p *= 7.4 × 10^-4^), but was close. Note that the information content at this region is only 0.36, indicating that from this region only about one-third of the possible meiotic information has been extracted. Additional markers in this region would assist in increasing the meiotic information and making the estimates of allele sharing between siblings more accurate and less dependent upon the specification of marker allele frequencies.

## Discussion

No results for TRD reach either suggestive or significant linkage in the context of genome-wide studies [9]. This implies that the effects of loci demonstrating TRD, if present at all, may not be a major concern for genetic mapping studies. However, when the results from this TRD analysis are compared with the predominantly nonsignificant results from genome-wide linkage studies of complex diseases then there may be cause for concern about the effects of TRD. This is important since it is common that the 'best' result from a genome-wide linkage scan for a complex disease is often followed-up with additional work, irrespective of whether that locus produces formally significant linkage.

Despite the relatively weakly significant results from this study, there are some observations worthy of comment. First, of the published genome-wide linkage studies of disease-related traits in the Framingham Heart Study families, the only trait-locus pair to produce significant linkage was mean systolic blood pressure to chromosome 17 at markers GATA25A04 and ATC6A06 [10]. In that study the pair-wise LOD scores for systolic blood pressure were 3.8 and 3.1 for these two markers respectively, and the multipoint LOD in that region reached 4.7. It is interesting to note that there is weak evidence for linkage of female sibships to the same markers (LOD = 1.82, *p *= 0.0017, Table 2). Evidence for linkage to the same region of these two traits (mean systolic blood pressure and female gender) could be purely coincidental, or could indicate either confounding or pleiotropy.

The region on chromosome 10 also deserves some mention (Table 3). Using data from CEPH families, we have previously observed excess sharing of alleles [3] on the short arm of chromosome 10. However, there are some important differences between the observations in that and this study. First, the region identified in the current analyses is much closer to the p-telomere (at 14 cM) than that identified previously (at 48 cM) according to the sex-averaged genetic map. Second, the region identified in the previous study was female-specific, whereas in the current study it was identified in the non-sex-specific analysis (Table 3), and results in the sex-specific analysis did not reach *p *< 0.002. This means that the current observation is certainly not a confirmation of our previous results. However, the mechanism(s) of TRD at any locus are generally unknown. Complex modes of inheritance for TRD have been described that at some loci depend upon the grandparental and parental origins of alleles in addition to the gender of the offspring [4,5]. Such complex modes of inheritance could not be tested in the Framingham data since there are too few three-generation families available for separate analysis.

Finally, families selected for large sibship size may actually bias against the detection of TRD loci. The reason for this is that families with large sibships will typically have fewer spontaneous abortions due to any TRD loci than families with small sibships. Therefore the parents of large sibships are expected to have a lower frequency of TRD-causing alleles than parents of families with fewer offspring. One implication of this could be that there is lower power to detect TRD loci in families with large sibships compared to those with smaller sibships. This concern applies to the CEPH reference families that have been used in some TRD studies [3-5] because they were recruited based upon large sibship size.

## Conclusions

In summary, we provide some evidence for TRD loci in families from the Framingham Heart Study. Although the effects observed are not significant, they could potentially complicate the mapping of complex disease loci.

## Figures and Tables

**Table 1:** Number and size of families.

**Families**	**2 Sibs**	**3 Sibs**	**4 Sibs**	**5 or More Sibs**	**Total Number of Families**
Female	166	43	11	2	222
Male	141	47	9	4	201
Not divided by sex	194	163	67	35	459

**Table 2:** Multipoint results for female and male sibships.

			**Female**			**Male**		
				
**Chr**	**Nearest Marker(s)**	**Position (cM)^A^**	**LOD**	***p***	**Info^B^**	**LOD**	***p***	**Info**
2	GATA52A04	200	0.07	0.70	0.85	1.93	0.0011	0.80
4	GATA27G03	168	0.00	0.42	0.77	1.86	0.0013	0.64
17	GATA25A04-ATC6A06	65	1.82	0.0017	0.80	0.59	0.0370	0.83
17	GATA49C09-ATA43A10	86	0.61	0.0420	0.57	1.77	0.0016	0.36

^A^Position according to the Marshfield map. ^B^Info, information content.

**Table 3:** Multipoint results in all families (not subdivided by gender).

**Chr**	**Nearest Marker(s)**	**Position (cM)^A^**	**LOD**	***p***	**Info^B^**
10	GATA88F09-AFM063xf4	14	2.05	7.5 × 10^-4^	0.36
20	AFM046xf6	96	1.10	0.0087	0.52
22	GATA11B12-GGAT3C10	41	1.75	0.0016	0.56

^A^Position according to the Marshfield map. ^B^Info, information content.
